# Supplementary material for: Treatment of peanut allergy and colitis in mice via the intestinal release of butyrate from polymeric micelles
Source: Nat Biomed Eng. 2022 Dec 22;7(1):38–55. doi: 10.1038/s41551-022-00972-5 (PMC9870785; doi:10.1038/s41551-022-00972-5)
Supplement: Supplementary file 1 — Supplementary figures and tables. [file 41551_2022_972_MOESM1_ESM.pdf]

nature biomedical engineering

Article https://doi.org/10.1038/s41551-022-00972-5

Treatment of peanut allergy and colitis in mice via the intestinal release of butyrate from polymeric micelles

In the format provided by the authors and unedited

Contents

Scheme 1 | Synthesis of N-(2-hydroxyethyl) methacrylamide (HEMA).
Scheme 2 | Synthesis of N-(2-butanoyloxyethyl) methacrylamide (BMA).
Scheme 3 | Synthesis of poly(2-hydroxypropyl methacrylamide) (pHPMA).
Scheme 4 | Synthesis of pHPMA-*b*-pBMA.
Scheme 5 | Synthetic route of pMAA-*b*-pBMA**.**
Scheme 6 | Synthesis of hydrophilic azide monomer N_3-PEG_4-MA.
Scheme 7 | Synthesis of hydrophobic control monomer N-hexyl methacrylamide.
Supplementary Fig. 1 | ^{1}H-NMR (500 MHz, $CDCl_3$) of HEMA.
Supplementary Fig. 2 | ^{1}H-NMR (500 MHz, $CDCl_3$) of BMA.
Supplementary Fig. 3 | ^{1}H-NMR (500 MHz, DMSO-d6) of pHPMA.
Supplementary Fig. 4 | ^{1}H-NMR (500 MHz, DMSO-d6) of pHPMA-*b*-pBMA.
Supplementary Fig. 5 | ^{1}H-NMR (500 MHz, DMSO-d6) of pMAA.
Supplementary Fig. 6 | ^{1}H-NMR (500 MHz, DMSO-d6) of pMAA-*b*-pBMA.
Supplementary Fig. 7 | ^{1}H-NMR (500 MHz, $CDCl_3$) of N_3-PEG_4-MA.
Supplementary Fig. 8 | ^{1}H-NMR (500 MHz, $CDCl_3$) of N-hexyl methacrylamide.
Supplementary Fig. 9 | ^{1}H-NMR (500 MHz, DMSO-d6) of control polymer pHPMA-*b*-pHMA.
Supplementary Fig. 10 | Size distribution of NtL-ButM and Neg-ButM.
Supplementary Fig. 11 | Critical micelle concentrations (CMC) of NtL-ButM and Neg-ButM.
Supplementary Fig. 12 | Small-angle X-ray scattering (SAXS) characterization of NtL-ButM and Neg-ButM.
Supplementary Fig. 13 | The DLS data of diameter distribution (by intensity) of micelles NtL-ButM and Neg-ButM in PBS or simulated gastric fluid (SGF).
Supplementary Fig. 14 | Derivatization of butyrate for LC-MS/MS analysis.
Supplementary Fig. 15 | Stability of pHPMA-b-pBMA polymer *in vitro* or *in vivo.*
Supplementary Fig. 16 | The biodistribution of NtL-ButM or Neg-ButM in the gastrointestinal (GI) tract of non-antibiotic treated SPF mice.
Supplementary Fig. 17 | NtL-ButM does not increase gene expression of defensins in SPF mice**.**
Supplementary Fig. 18 | Differentially abundant taxa within each treatment group before and after two-week treatment with PBS or ButM as analyzed by LEfSe from **Fig. 6**.
Supplementary Fig. 19 | Representative gating strategy for identifying $Foxp3^+CD25^+$ $CD4^+$ T cells present in spleen.
Supplementary Fig. 20 | Representative gating strategy for identifying $CD11c^{hi}$, $CD11b^+F4/80^+$, and $CD11b^+CD11c^-$ in the mesenteric LNs, and the $MHCII^+$ or $CD86^+$ cells in those cell subsets.
Supplementary Table 1 | Primer sequences for qPCR.

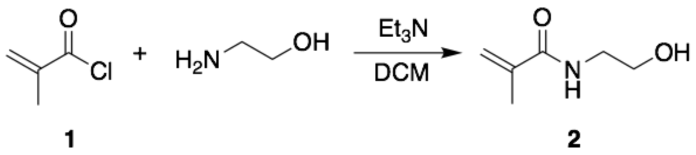

Scheme 1 | Synthesis of N-(2-hydroxyethyl) methacrylamide (HEMA, **2**)

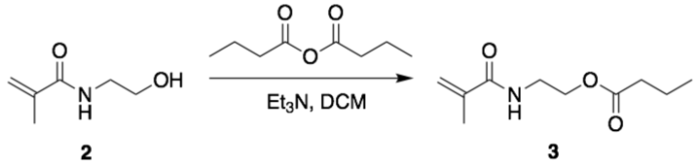

Scheme 2 | Synthesis of N-(2-butanoyloxyethyl) methacrylamide (BMA, **3**)

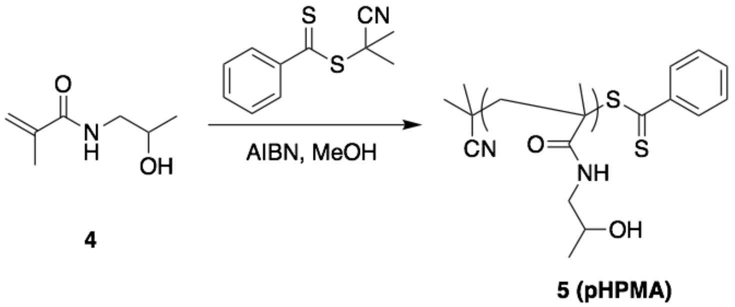

Scheme 3 | Synthesis of poly(2-hydroxypropyl methacrylamide) (pHPMA, **5**)

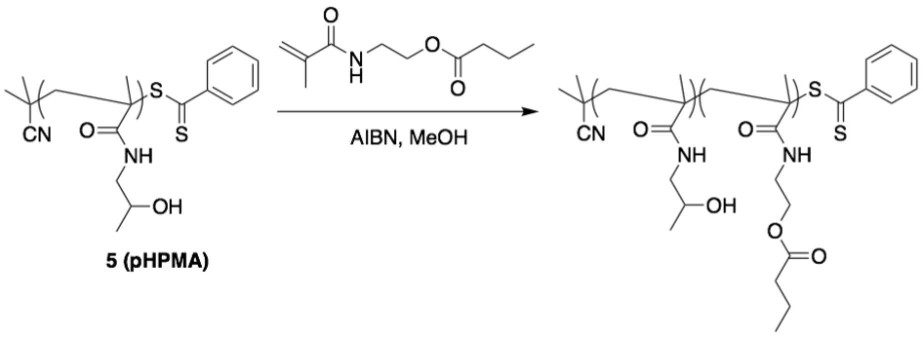

6 (pHPMA-b-pBMA)

Scheme 4 | Synthesis of pHPMA-*b*-pBMA (**6**)

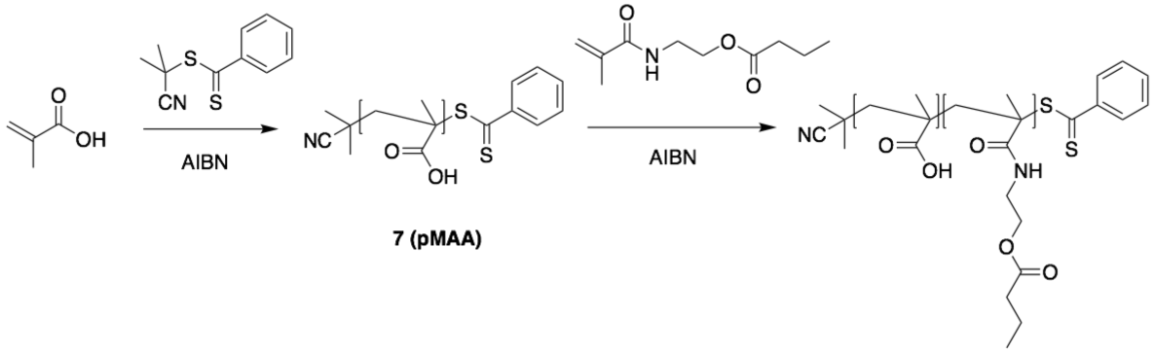

8 (pMAA-*b*-pBMA)

Scheme 5 | Synthetic route of pMAA-*b*-pBMA (**8**)

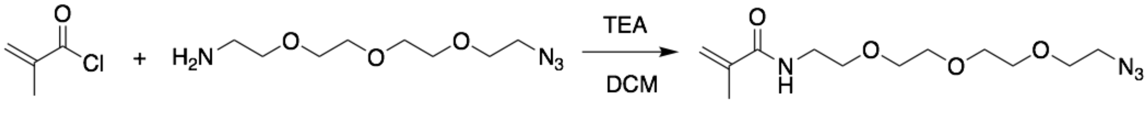

9 N_3-PEG$_4$-MA

Scheme 6 | Synthesis of hydrophilic azide monomer N_3-PEG$_4$-MA (**9**)

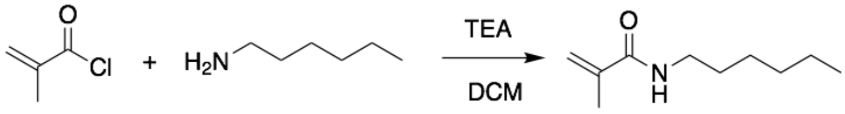

10

Scheme 7 | Synthesis of hydrophobic control monomer N-hexyl methacrylamide (**10**)

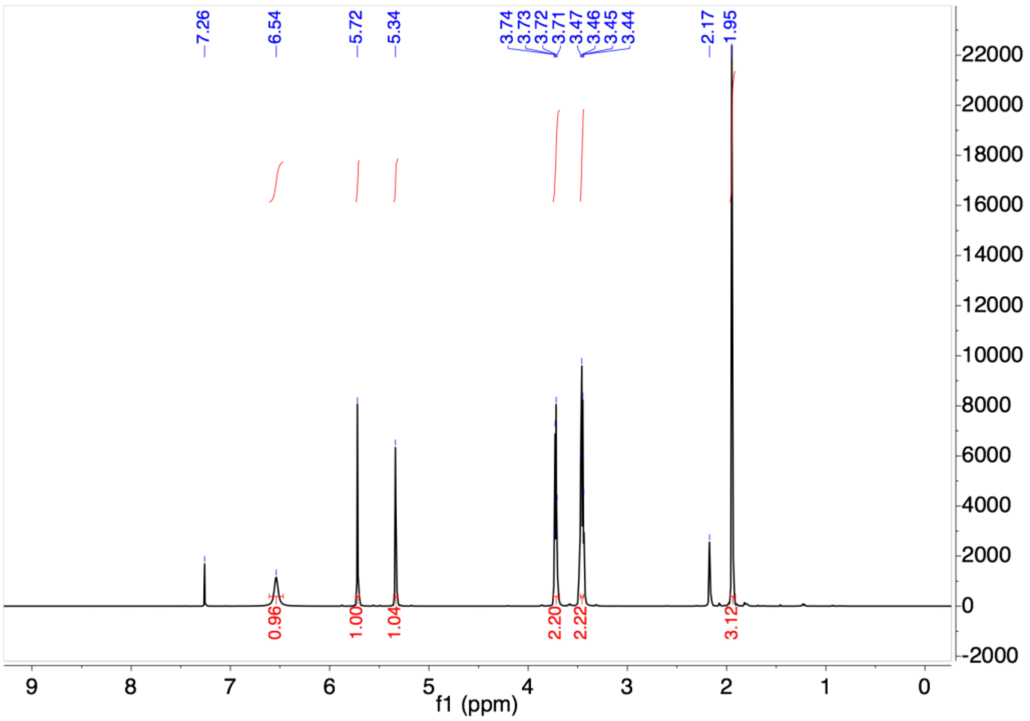

Supplementary Fig. 1 | ^{1}H-NMR (500 MHz, $CDCl_3$) of HEMA (**2**)

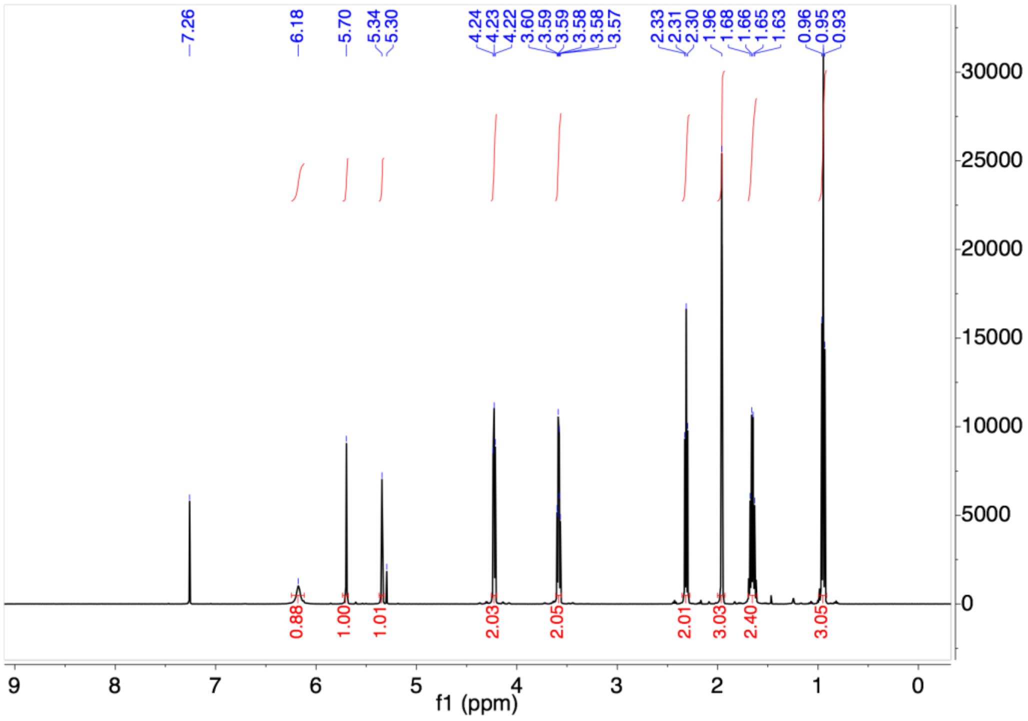

Supplementary Fig. 2 | ^{1}H-NMR (500 MHz, $CDCl_3$) of BMA (**3**)

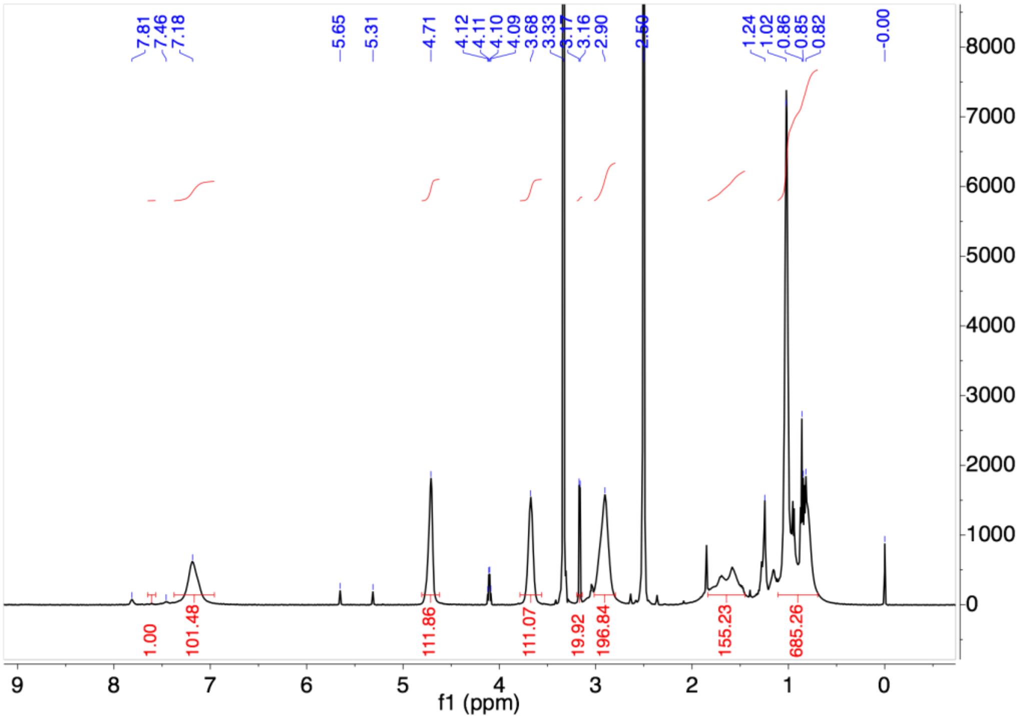

Supplementary Fig. 3 | ^{1}H-NMR (500 MHz, DMSO-d6) of pHPMA (**5**)

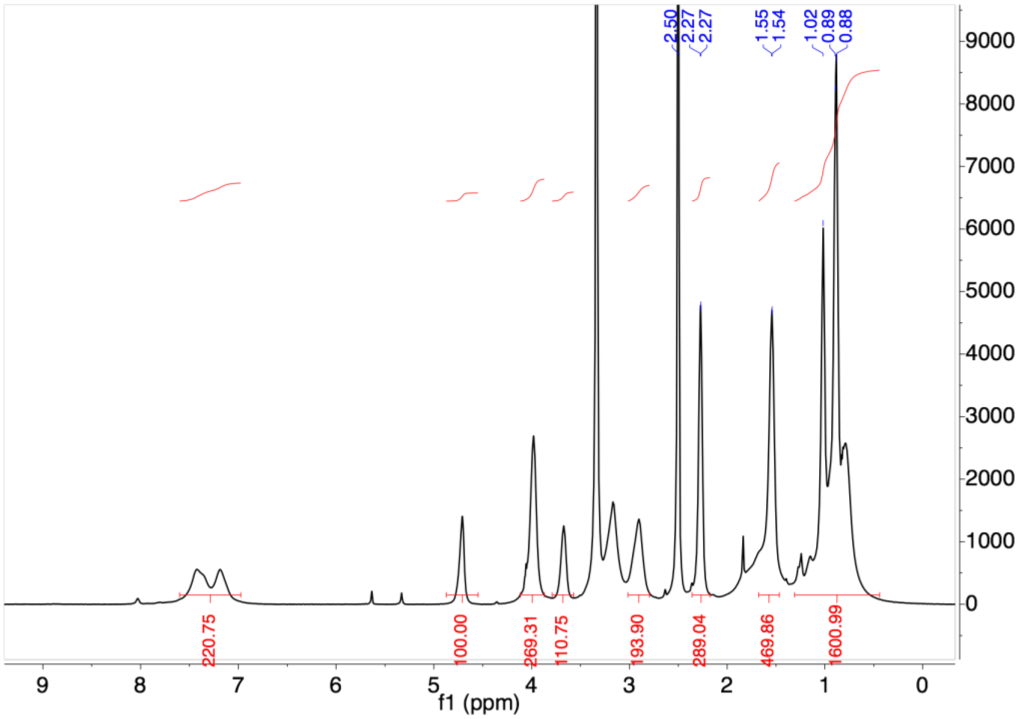

Supplementary Fig. 4 | ^{1}H-NMR (500 MHz, DMSO-d6) of pHPMA-*b*-pBMA (**6**)

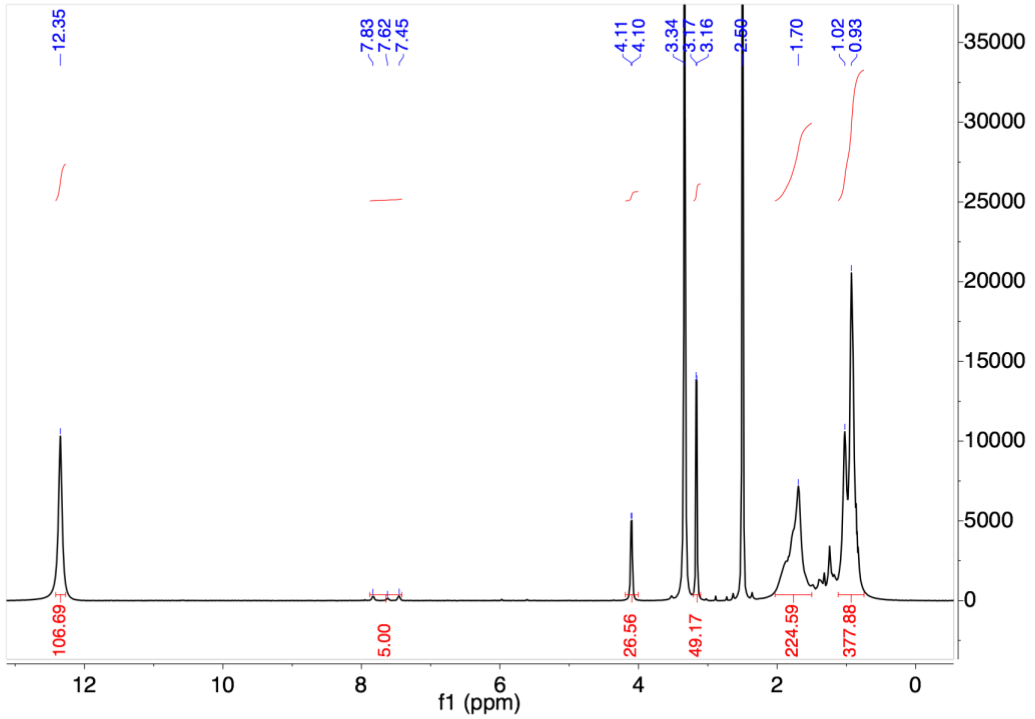

Supplementary Fig. 5 | ^{1}H-NMR (500 MHz, DMSO-d6) of pMAA (**7**)

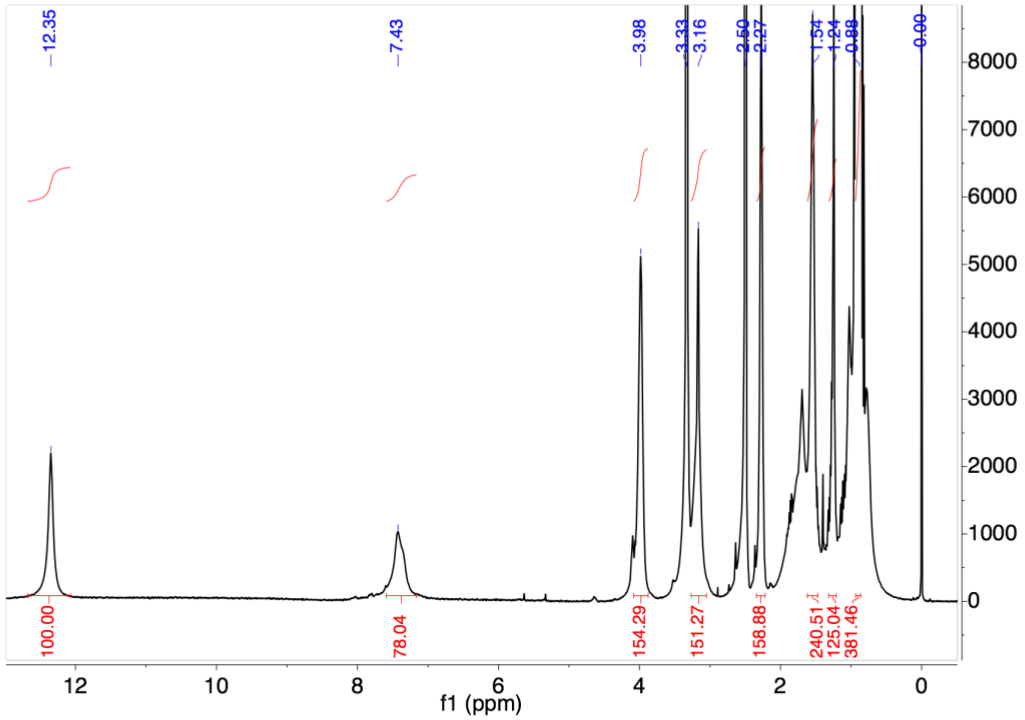

Supplementary Fig. 6 | ^{1}H-NMR (500 MHz, DMSO-d6) of pMAA-*b*-pBMA (**8**)

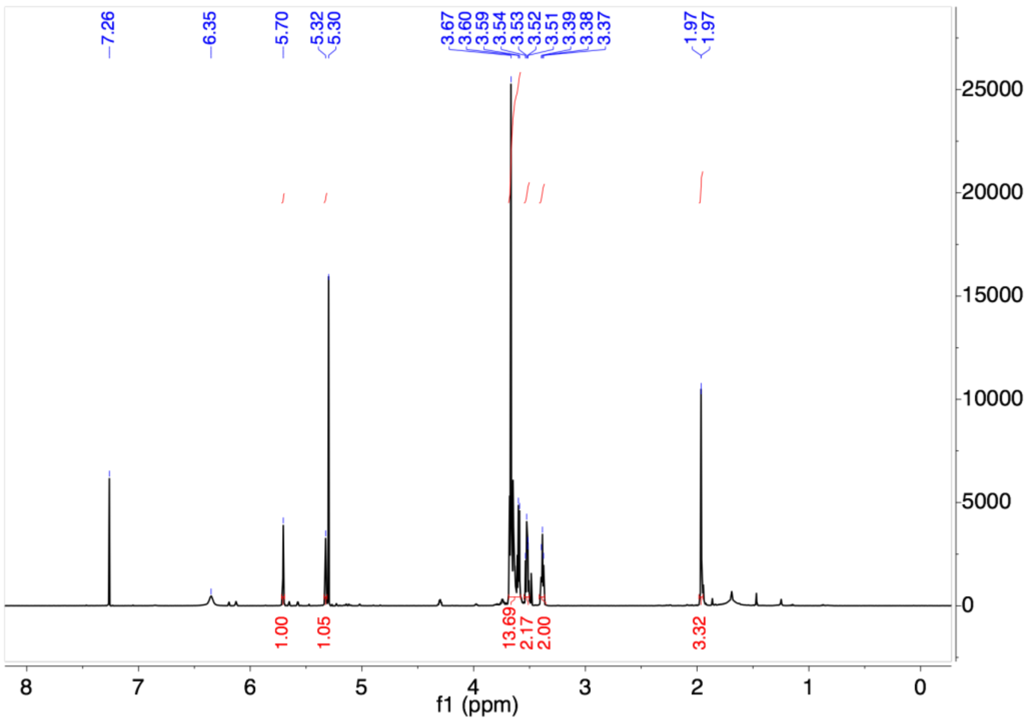

Supplementary Fig. 7 | ^{1}H-NMR (500 MHz, $CDCl_3$) of N_3-PEG$_4$-MA (**9**)

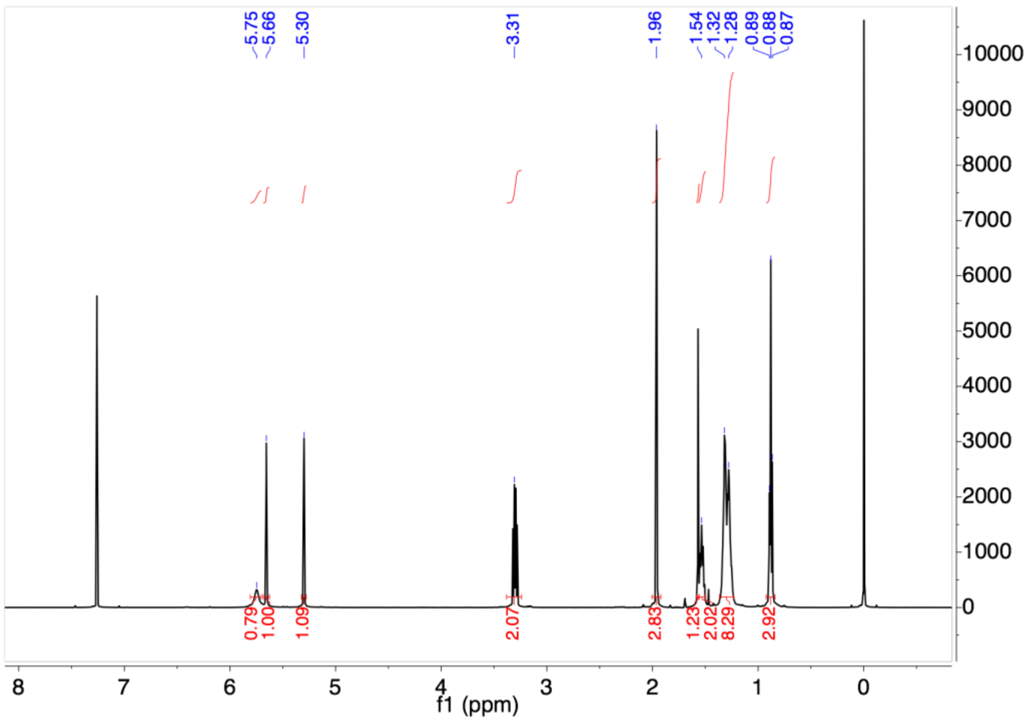

Supplementary Fig. 8 | ^{1}H-NMR (500 MHz, $CDCl_3$) of N-hexyl methacrylamide (**10**)

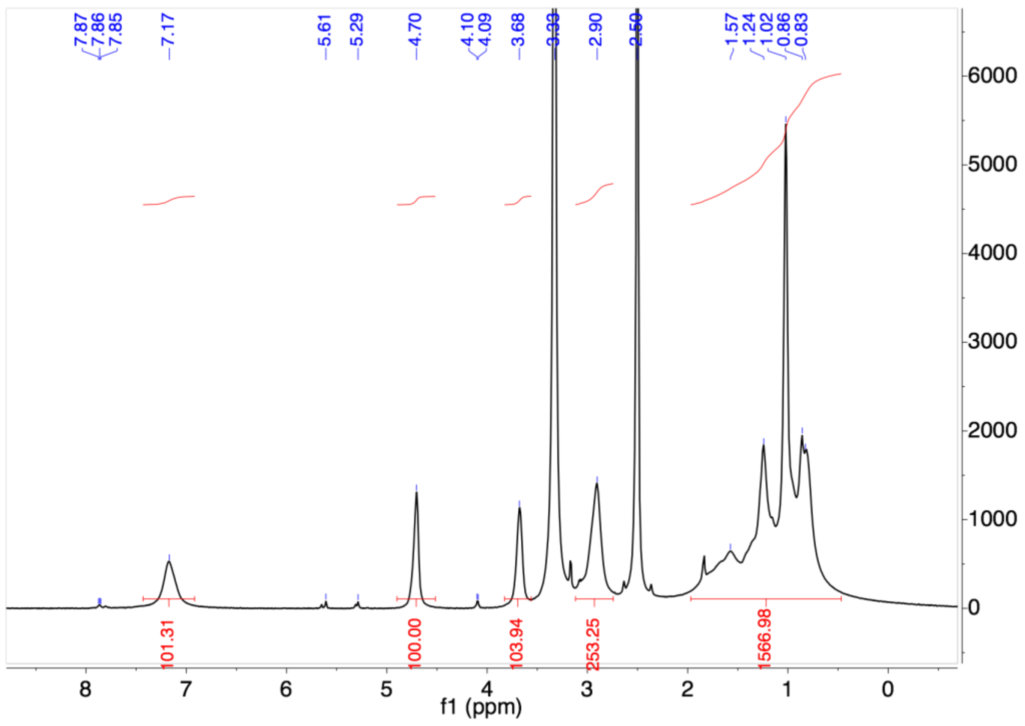

Supplementary Fig. 9 | ^{1}H-NMR (500 MHz, DMSO-d6) of control polymer pHPMA-*b*-pHMA

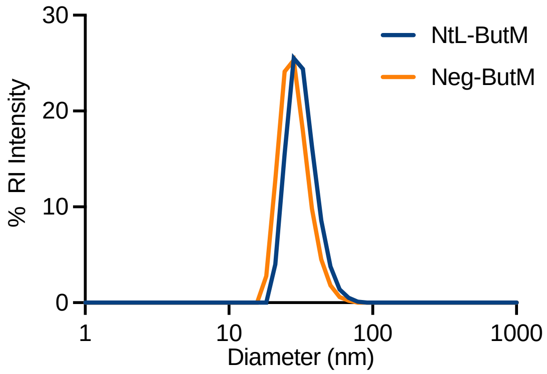

Supplementary Fig. 10 | Dynamic light scattering (DLS) shows that the micelles NtL-ButM and Neg-ButM have similar hydrodynamic diameters, at sub-hundred nanometer.

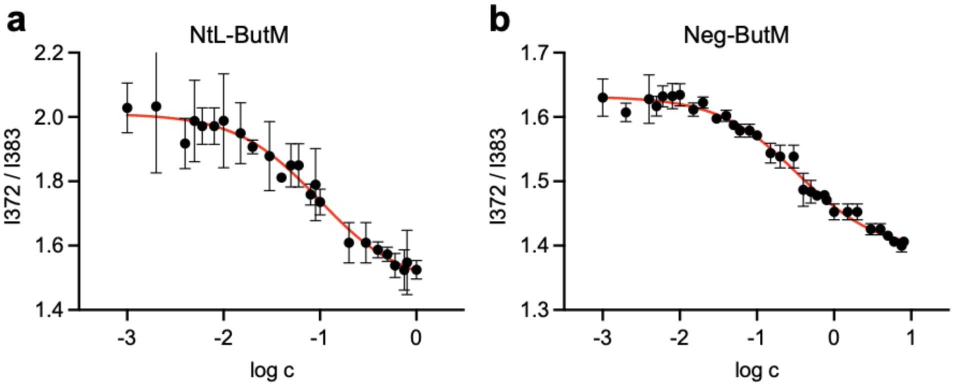

Supplementary Fig. 11 | Critical micelle concentrations (CMC) of NtL-ButM (**a**) and Neg-ButM (**b**) measured by pyrene fluorescent intensity of peak 1 over peak 3. The CMC was determined by the IC50 fitted by a sigmoidal curve.

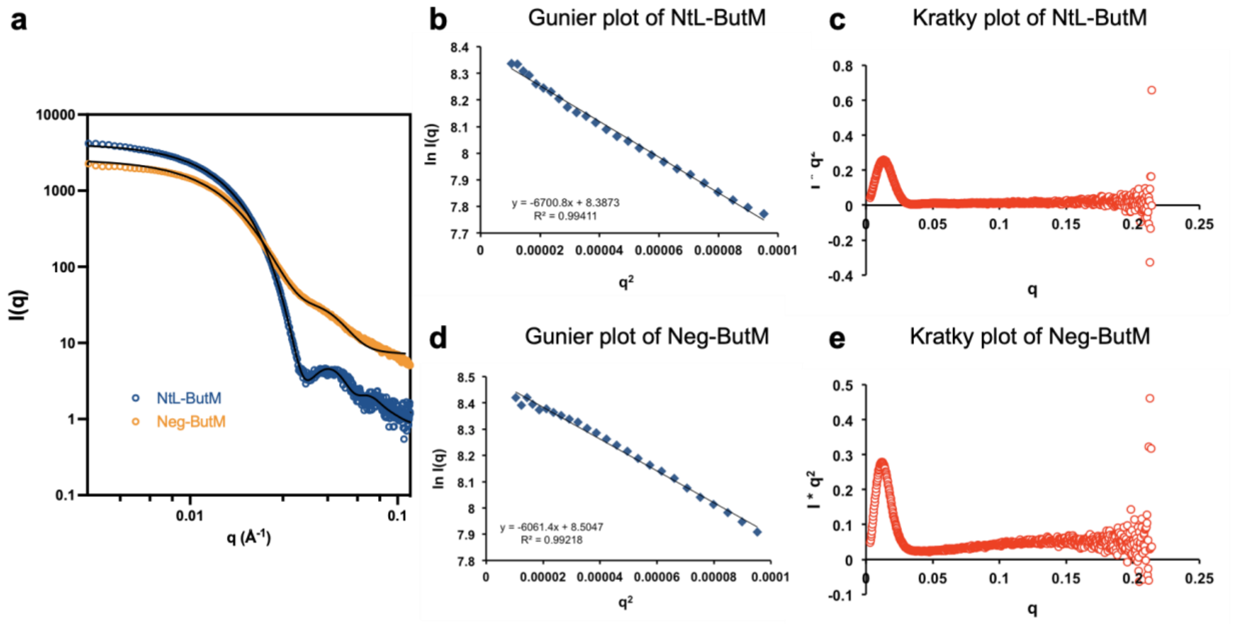

f

	c (mg mL^{-1})	radius$_{core}$ (Å)	thickness$_{shell}$ (Å)	SLD$_{core}$ (10^{-6} Å^{-2})	SLD$_{shell}$ (10^{-6} Å^{-2})	volume fraction
NtL-ButM	2.0	119.31 ± 0.16	72.63 ± 0.31	16.1821 ± 0.0016	10.5338 ± 0.0003	0.007381
Neg-ButM	2.0	83.84 ± 0.34	98.97 ± 0.22	16.9295 ± 0.0023	10.8700 ± 0.0004	0.01393

g

	c (mg mL^{-1})	d (nm)	N ($\times 10^{14}$ mL^{-1})	Mw ($\times 10^{6}$ g mol^{-1})	N_{agg}
NtL-ButM	2.0	158.9	2.492	4.831	**119**
Neg-ButM	2.0	122.5	5.441	2.213	**92**

Supplementary Fig. 12 | Small-angle X-ray scattering (SAXS) characterization of NtL-ButM and Neg-ButM micelles. **a**, SAXS data of NtL-ButM and Neg-ButM. Data are fitted with polydisperse core-shell model. **b**, Gunier plot (ln(q) vs. q^2) of NtL-ButM revealed the radius of gyration of the micelle. **c,** Kratky plot ($I\ q^2$ vs. q) of NtL-ButM revealed the spherical structure if the micelle. **d,** Gunier plot of Neg-ButM micelle. **e**, Kratky plot of Neg-ButM micelle. **f**, Table of fitting parameters of NtL-ButM and Neg-ButM using a polydisperse core-shell sphere model. **g,** Table of the mean distance between micelles d, number of micelles per unit volume N, molecular weight of the micelle Mw, and the aggregation number N_{agg}, calculated from the fitting parameters of a polydisperse core-shell sphere model.

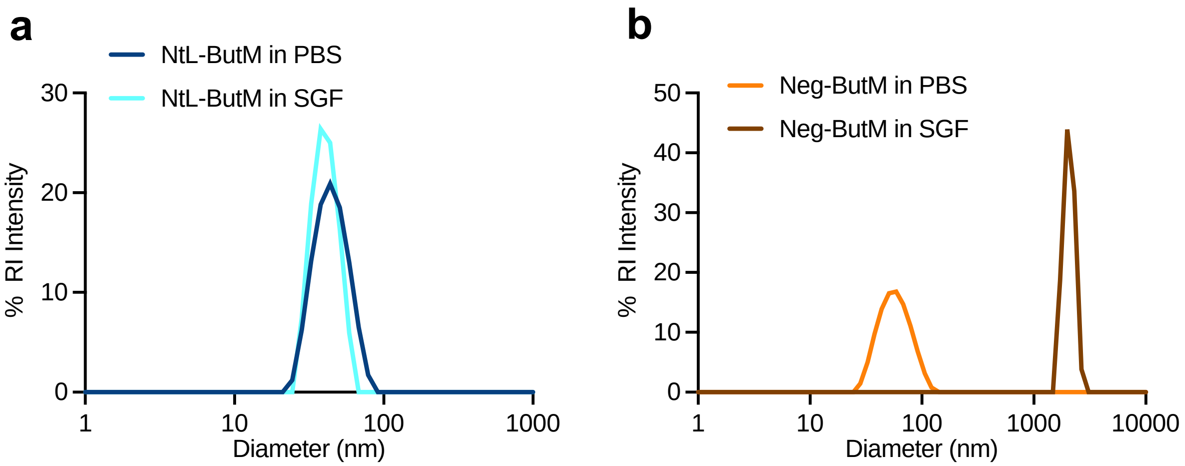

Supplementary Fig. 13 | The DLS data of diameter distribution (by intensity) of micelles NtL-ButM (**a**) and Neg-ButM (**b**) in PBS or simulated gastric fluid (SGF).

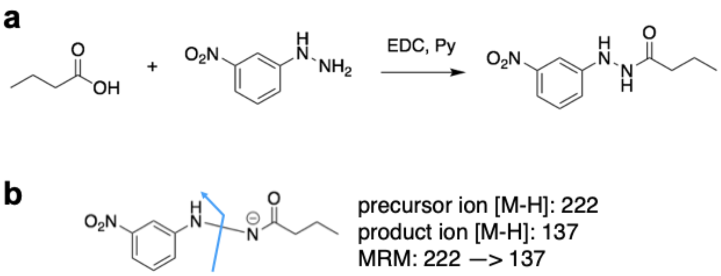

Supplementary Fig. 14 | Derivatization of butyrate for LC-MS/MS analysis and the release of butyrate from NtL-ButM/Neg-ButM in simulated gastric/intestinal fluids. **a,** Derivatization reaction of butyrate with 3-nitrophenylhydrazine (NPH) to generate UV active butyrate-NPH. **b,** The multiple reaction monitoring (MRM) of 222 → 137 was used to quantify butyrate-NPH in LC-MS/MS.

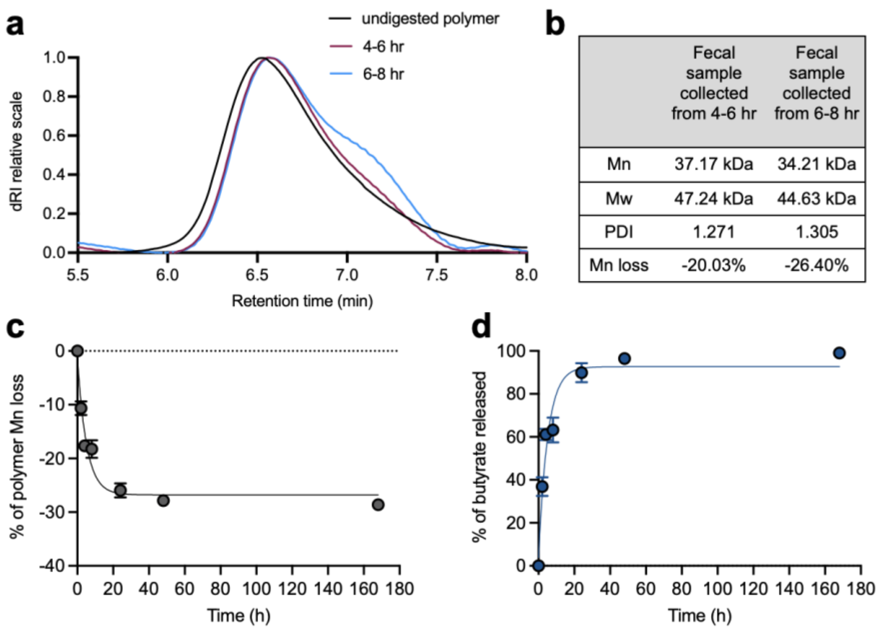

	Fecal sample collected from 4-6 hr	Fecal sample collected from 6-8 hr
Mn	37.17 kDa	34.21 kDa
Mw	47.24 kDa	44.63 kDa
PDI	1.271	1.305
Mn loss	-20.03%	-26.40%

Supplementary Fig. 15 | Stability of pHPMA-b-pBMA polymer *in vitro* or *in vivo*. **a**, Gel permeation chromatography (GPC) elution profiles (measured by differential refractive index (dRI) over time) of polymers collected from pooled fecal samples of two mice treated with NtL-ButM at 4-6 hr (red) or 6-8 hr (blue) post-gavage. Black curve: polymer control. **b,** The table of molecular weight of digested polymer measured by GPC, including the number averaged molecular weight (Mn) and weight averaged molecular weight (Mw), with their polydispersity index (PDI) and the Mn loss compared to undigested polymer control. **c, d,** The Mn loss of pHPMA-b-pBMA polymer in 125 mM NaOH solution over 7 days (**c**), or the percentage of butyrate released from the polymer (**d**), measured by GPC, n=3, Data represent mean ± s.e.m.

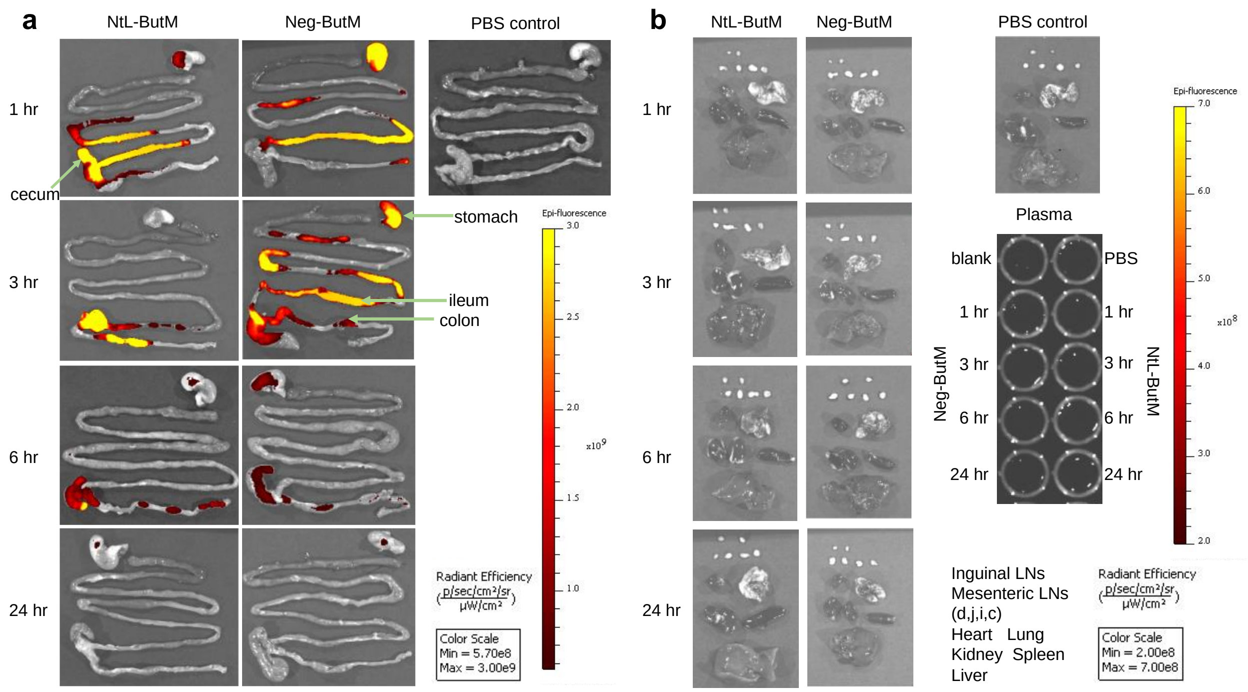

Supplementary Fig. 16 | The biodistribution of NtL-ButM or Neg-ButM in the gastrointestinal (GI) tract of non-antibiotic treated SPF mice. Mice were administered IR750 labeled NtL-ButM or Neg-ButM intragastrically and euthanized at the indicated time points to measure the fluorescent signals in the GI tract (**a**), and other major organs and plasma (**b**) using an in vivo imaging system (IVIS). Both polymers were chemically modified with azide and labeled with dye IR750. After a single oral administration of labeled NtL-ButM or Neg-ButM (one mouse per time point per treatment group), IVIS showed Neg-ButM retained in the stomach for more than 6 hr while NtL-ButM moved to the cecum quickly. Both polymers were cleared from the GI tract after 24 hr, and there was no absorption of either butyrate micelle into the systemic circulation. Mesenteric LNs (d, duodenum-draining; j, jejunum-draining; I, ileum-draining; c, colon-draining).

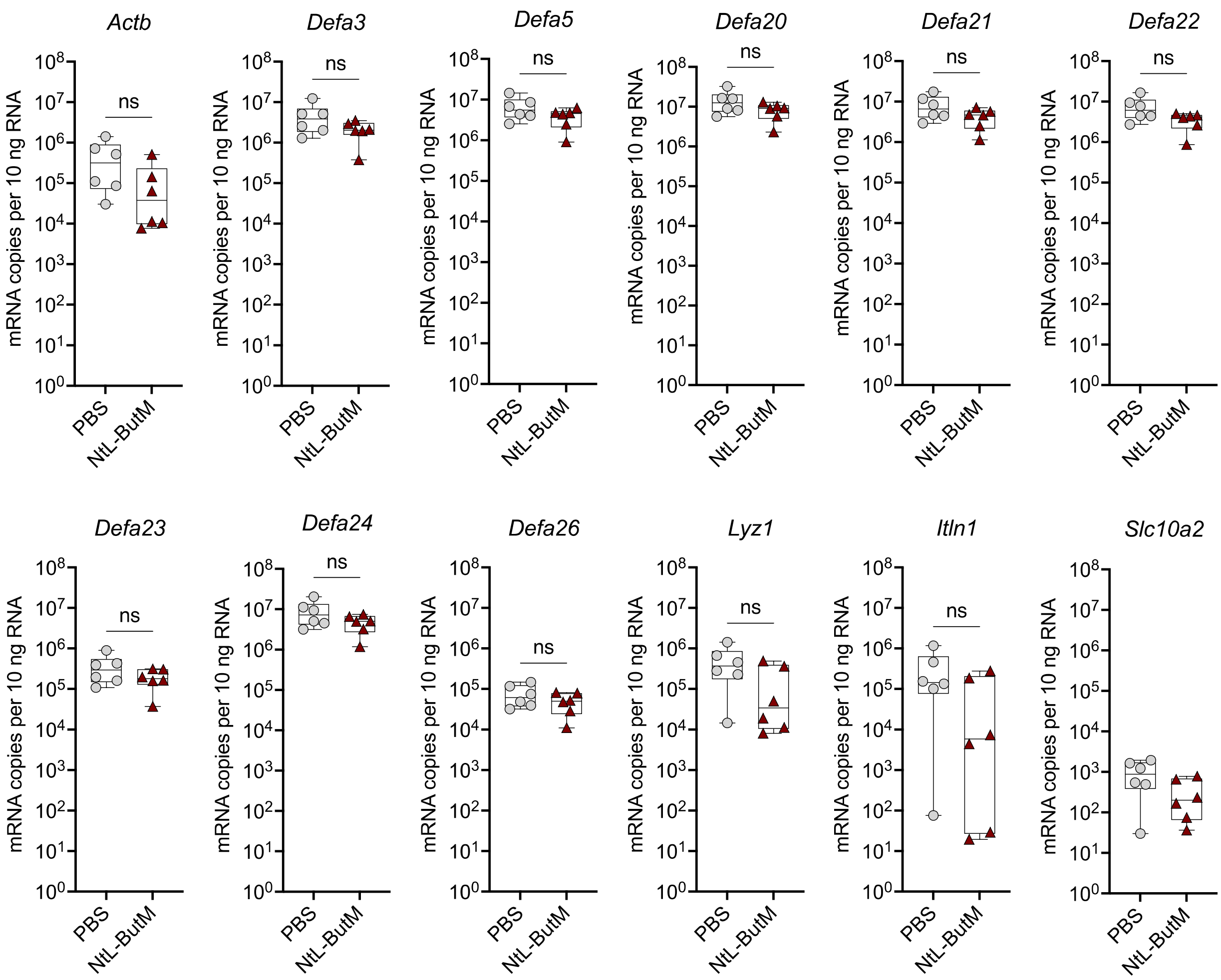

Supplementary Fig. 17 | NtL-ButM does not increase gene expression of defensins in SPF mice**.** Differentially expressed genes (DEGs) in the ileum of 8-week-old SPF C57BL/6 mice that were treated daily with 800 mg kg^{-1} NtL-ButM (n = 6) for one week, compared to PBS-treated controls (n = 6) as measured by RT-qPCR of ileal epithelial cells. Data represent mean ± s.e.m. Data analyzed using two-sided Student's t-test.

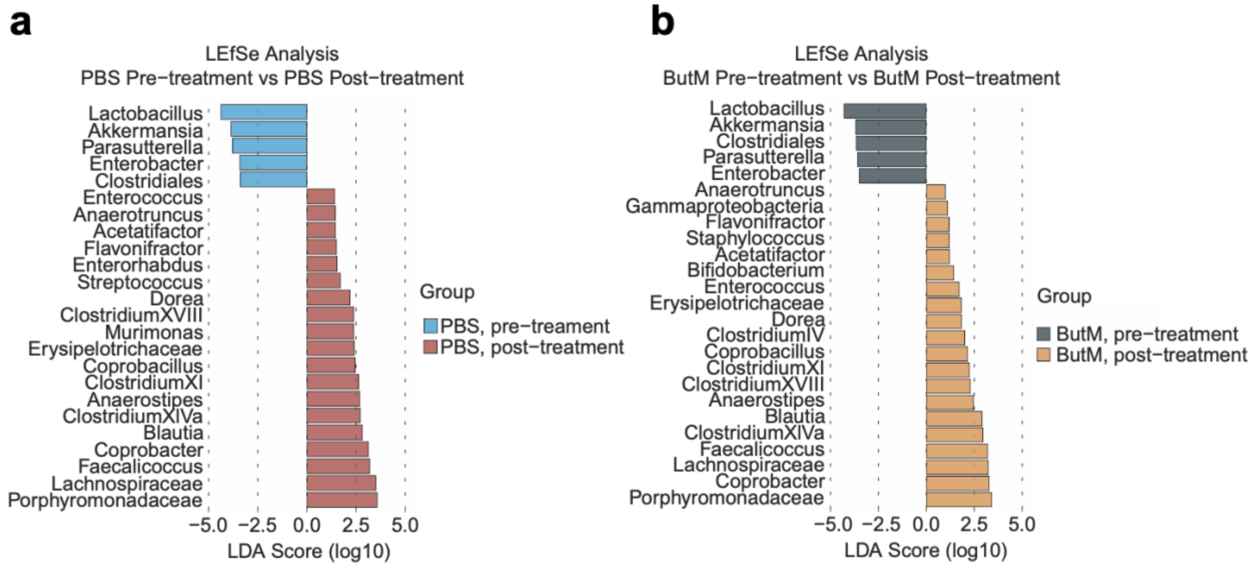

Supplementary Fig. 18 | Differentially abundant taxa within each treatment group before and after two-week treatment with PBS (**a**) or ButM (**b**) as analyzed by LEfSe from **Fig. 6**.

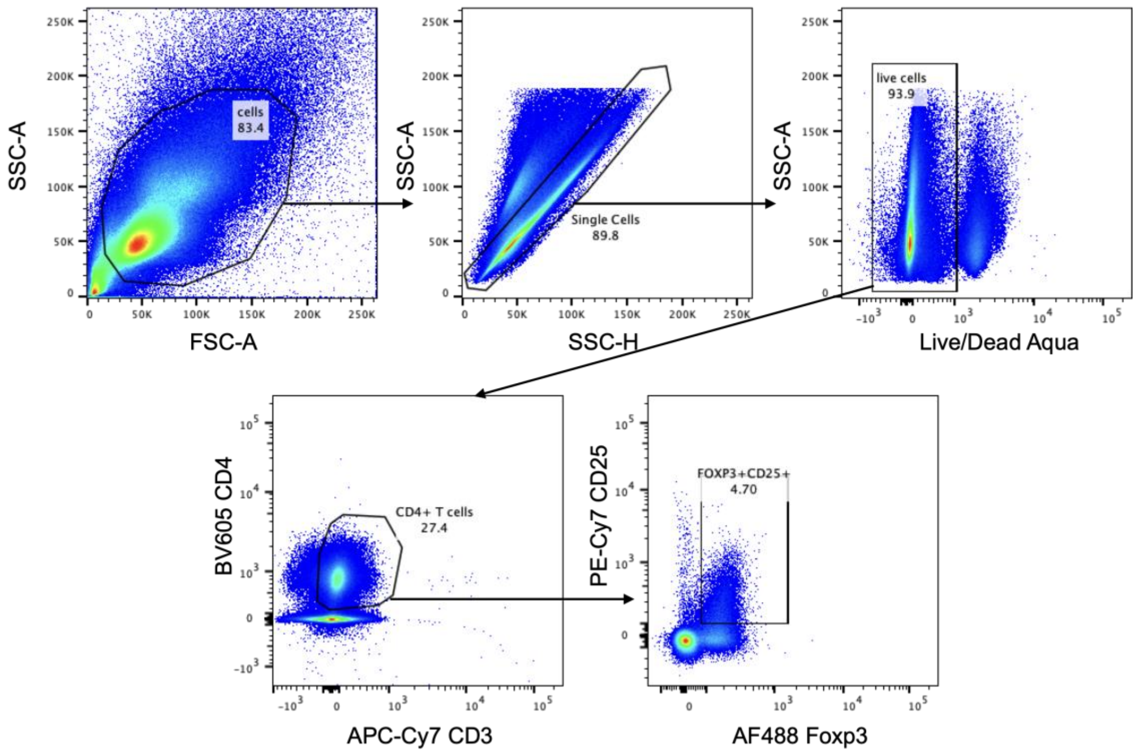

Supplementary Fig. 19 | Representative gating strategy for identifying Foxp3^{+}CD25^{+} CD4^{+} T cells present in spleen.

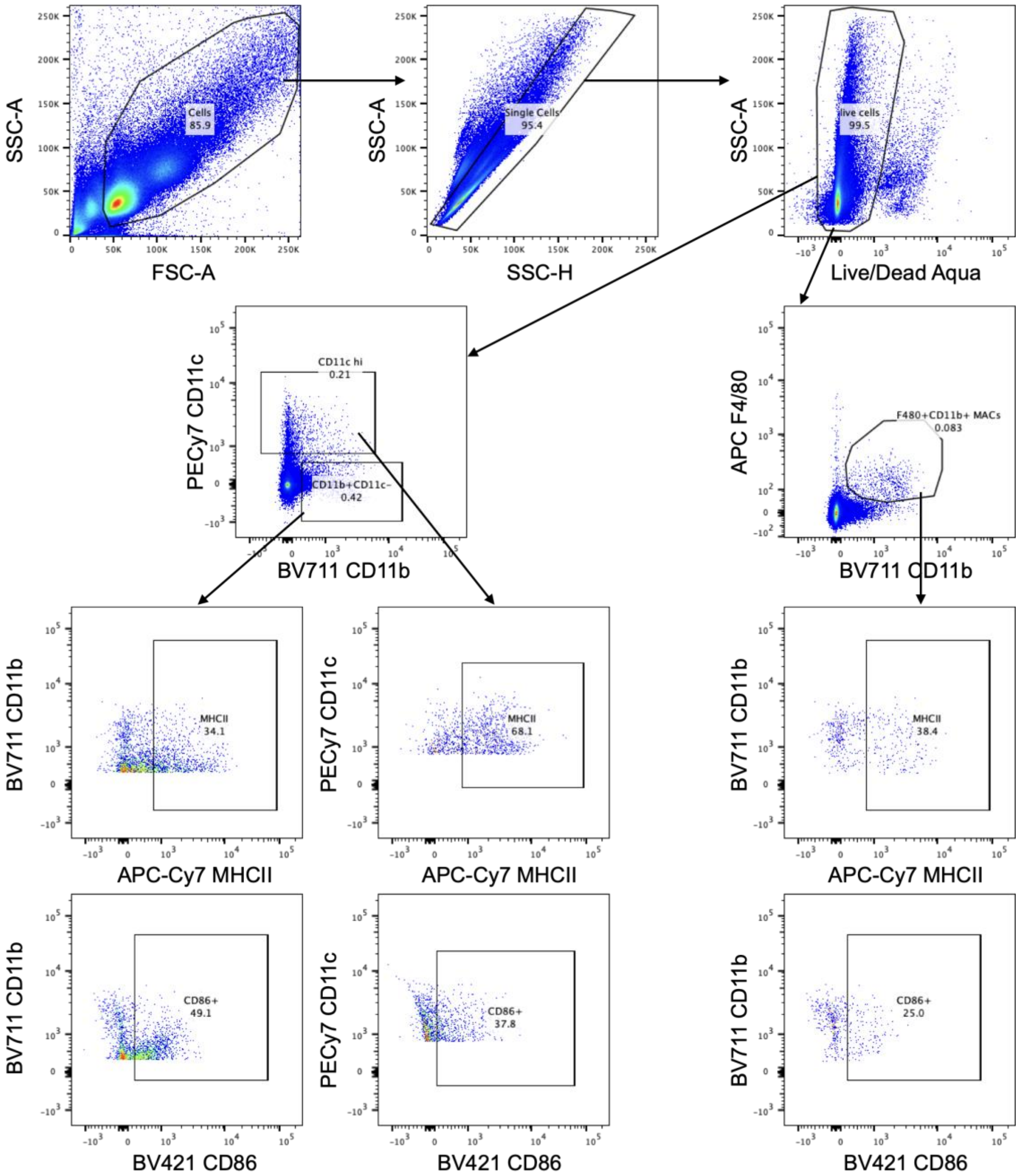

Supplementary Fig. 20 | Representative gating strategy for identifying CD11c^{hi}, CD11b^{+}F4/80^{+}, and CD11b^{+}CD11c^{-} in the mesenteric LNs, and the MHCII^{+} or CD86^{+} cells in those cell subsets.

Supplementary Table 1 | Primer sequences for qPCR.

Target	Primer sequence (5'->3')	Reference
Clostridium Cluster XIVa	Forward: AAATGACGGTACCTGACTAA	73
	Reverse: CTTTGAGTTTCATTCTTGCGAA	

Primers to quantify total bacterial load	Primer sequence (5'->3')	Reference
	8F: AGAGTTTGATCCTGGCTCAG	71
	338R: TGCTGCCTCCCGTAGGAGT	72

Target	Primer sequence (5'->3')	Reference
Actb	F: GGCTGTATTCCCCTCCATCG	
	R: CCAGTTGGTAACAATGCCATGT	65
Defa3	F: TCCTGCTCACCAATCCTCCAGGT	
	R: CATATTGCGAACAATTTATTG	42
Defa5	F: TCCTGCTCAACAATTCTCCAG	
	R: CATATTGCAAACAATTTATTG	42
Defa20	F: TCCTGCTCAACAATTCTCCAG	
	R: CATATTGCAAACAATTTATTG	42
Defa21	F: TCCTGCTCACCAATCCTCCAGGT	
	R: CATATTGCAAACAATTTATTG	42
Defa22	F: TCCTGCTCACCAATCCTCCAGGT	
	R: CATATTGCAAACAATTTATTG	42
Defa23	F: TCCTGCTCACCAATCCTCCAGGT	
	R: CATATTGCGAACAATTTATTG	42
Defa24	F: TGCTACTCACCAATCCTCCAGGT	
	R: CATATTGCAAGCAATTTATTG	42
Defa26	F: TCCTGCTCCCCAATCCCCCAGGT	
	R: CATATTGCGGACAATTTATTG	42
Lyz1	F: 5′-AAAACCCCAGGAGCAGTTAAT-3′	
	R: 5′-CAACCCTCTTTGCACAAGCT-3′	62
Itln1	F: '5'- ACCGCACCTTCACTGGCTTC-3'	
	R: 5'- CCAACACTTTCCTTCTCCGTATTTC-3'	65
Slc10a2	F: TTGCCTCTTCGTCTACACC	
	R: CCAAAGGAAACAGGAATAACAAG	42
