# Supplementary material for: Treatment of peanut allergy and colitis in mice via the intestinal release of butyrate from polymeric micelles
Source: Nat Biomed Eng. 2022 Dec 22;7(1):38–55. doi: 10.1038/s41551-022-00972-5 (PMC9870785; doi:10.1038/s41551-022-00972-5)
Supplement: Supplementary file 2 — Reporting Summary [file 41551_2022_972_MOESM2_ESM.pdf]

nature portfolio

Corresponding author(s): Cathryn R. Nagler,Jeffrey A. Hubbell

Last updated by author(s): November 27, 2022

Reporting Summary

Nature Portfolio wishes to improve the reproducibility of the work that we publish. This form provides structure for consistency and transparency in reporting. For further information on Nature Portfolio policies, see our Editorial Policies and the Editorial Policy Checklist.

Statistics

For all statistical analyses, confirm that the following items are present in the figure legend, table legend, main text, or Methods section.

n/a	Confirmed	
☐	☒	The exact sample size (*n*) for each experimental group/condition, given as a discrete number and unit of measurement
☐	☒	A statement on whether measurements were taken from distinct samples or whether the same sample was measured repeatedly
☐	☒	The statistical test(s) used AND whether they are one- or two-sided<br>*Only common tests should be described solely by name; describe more complex techniques in the Methods section.*
☐	☒	A description of all covariates tested
☒	☐	A description of any assumptions or corrections, such as tests of normality and adjustment for multiple comparisons
☐	☒	A full description of the statistical parameters including central tendency (e.g. means) or other basic estimates (e.g. regression coefficient) AND variation (e.g. standard deviation) or associated estimates of uncertainty (e.g. confidence intervals)
☐	☒	For null hypothesis testing, the test statistic (e.g. *F*, *t*, *r*) with confidence intervals, effect sizes, degrees of freedom and *P* value noted<br>*Give P values as exact values whenever suitable.*
☒	☐	For Bayesian analysis, information on the choice of priors and Markov chain Monte Carlo settings
☒	☐	For hierarchical and complex designs, identification of the appropriate level for tests and full reporting of outcomes
☒	☐	Estimates of effect sizes (e.g. Cohen's *d*, Pearson's *r*), indicating how they were calculated

Our web collection on statistics for biologists contains articles on many of the points above.

Software and code

Policy information about availability of computer code

Data collection	Bruker TopSpin (3.x) was used to collect NMR data. Agilent ChemStation was used to collect LCMS and Triple Quad MS-MS data. Wyatt Dynamics was used to collect DLS and zeta-potential data. Tosoh EcoSEC software or Empower Pro analysis software and Wyatt analysis software (ASTRA) was used to collect GPC data. Chromeleon CDS 7 was used to collect LC-UV data. FastQC (v0.11.5) was used to assess raw RNA sequencing data. LSR Fortessa flow cytometer (BD Biosciences) was used to collect flow cytometry data. Data collection for 16S rRNA targeted sequencing is detailed in the Methods session.
Data analysis	MNova (14.2.0) was used to analyze NMR data. Igor Pro 8 was used to analyze SAXS data. ImageJ (1.8.0) was used to analyze CryoEM data. Living Imaging 4.5.5 (Perkin Elmer) was used to analyze IVIS data. Fiji was used to analyze fluorescent images of intelectin staining. FlowJo 10.8.0 was used to analyze flow cytometry data. Data analysis for the RNA sequencing, 16S rRNA targeted sequencing, and microbiome analysis are detailed in the Methods session. Prism software (GraphPad v9) was used to conduct all statistical tests. Microsoft Excel was used to carry out simple operations.

For manuscripts utilizing custom algorithms or software that are central to the research but not yet described in published literature, software must be made available to editors and reviewers. We strongly encourage code deposition in a community repository (e.g. GitHub). See the Nature Portfolio guidelines for submitting code & software for further information.

March 2021

Data

Policy information about availability of data

All manuscripts must include a data availability statement. This statement should provide the following information, where applicable:

- Accession codes, unique identifiers, or web links for publicly available datasets
- A description of any restrictions on data availability
- For clinical datasets or third party data, please ensure that the statement adheres to our policy

The main data supporting the results in this study are available within the paper and its Supplementary Information. Additional processed data are available from the corresponding authors upon request. The 16S rRNA and RNAseq raw FastQ data files have been deposited into the National Center for Biotechnology (NCBI) Sequence Read Archive are available under accession numbers PRJNA863725 and PRJNA885957, respectively.

Field-specific reporting

Please select the one below that is the best fit for your research. If you are not sure, read the appropriate sections before making your selection.

☒ Life sciences ☐ Behavioural & social sciences ☐ Ecological, evolutionary & environmental sciences

For a reference copy of the document with all sections, see nature.com/documents/nr-reporting-summary-flat.pdf

Life sciences study design

All studies must disclose on these points even when the disclosure is negative.

Sample size	Sample size was determined using the results obtained from previous and preliminary studies. Each experiment contained 9-16 mice per group. See figure legends for details on n for each display figure.
Data exclusions	No data was excluded from the analysis.
Replication	At least 5, and in most cases 10, independent biological replicates were examined for each datapoint analyzed.
Randomization	Littermate controlled mice were bred in house and randomly allocated into each treatment. For experiments performed by Inotiv, mice were purchased from a vendor and randomly allocated into treatment groups.
Blinding	The person assessing the temperature for all challenges was a second person different from the person taking care of the mice and treatments and blinded to the treatment group. All mice were identified by a unique 5 digit ear-tag which allowed for blinding during ELISA assays and other analysis as results were first analyzed and tabulated based on the 5 digit identifiers before being matched to experimental groups. The person performing fluorescent imaging and image analysis was blinded to treatment group.

Reporting for specific materials, systems and methods

We require information from authors about some types of materials, experimental systems and methods used in many studies. Here, indicate whether each material, system or method listed is relevant to your study. If you are not sure if a list item applies to your research, read the appropriate section before selecting a response.

Materials & experimental systems

n/a	Involved in the study	
☐	☒	Antibodies
☒	☐	Eukaryotic cell lines
☒	☐	Palaeontology and archaeology
☐	☒	Animals and other organisms
☒	☐	Human research participants
☒	☐	Clinical data
☒	☐	Dual use research of concern

Methods

n/a	Involved in the study	
☒	☐	ChIP-seq
☐	☒	Flow cytometry
☒	☐	MRI-based neuroimaging

Antibodies

Antibodies used	Goat anti-mouse IgE UNLB Southern Biotech Cat# 1110-01 (1:2,000)<br>Rabbit anti-goat IgG (H+L) secondary antibody AP conjugate, Invitrogen, Cat# 31300, (1:5,000)<br>Goat anti-mouse IgG1-HRP conjugated, Southern Biotechnology Associates, Cat#1071-05<br>Mouse MCPT-1 Uncoated ELISA Kit: Thermofisher Scientific, Cat# 88-7503-88<br>Histamine EIA Kit, Oxford Biomedical Research, Cat#EA31

	Rat anti-mouse intelectin-1, R&D Systems, Cat# MAB8074<br>APC anti-mouse CD4, clone GK1.5, BioLegend, Cat#100412<br>FITC anti-CD45RB, clone GK1.5, BioLegend, Cat#100412<br>PE anti-CD25, clone GK1.5, BioLegend, Cat#100412<br>APC-Cy™7 Hamster Anti-Mouse CD3e, clone 145-2C11, BD Biosciences, Cat#557596<br>Brilliant Violet 605™ anti-mouse CD4, clone RM4-5, Biolegend, Cat#100547<br>PE/Cyanine7 anti-mouse CD25, clone PC61, Biolegend,Cat#102016<br>Alexa Fluor® 488 Rat anti-Mouse Foxp3, clone MF23, BD Biosciences, Cat#560407<br>BV711 Rat Anti-CD11b, clone M1/70, BD Biosciences, Cat#563168<br>PE-Cy™7 Hamster Anti-Mouse CD11c, clone HL3, BD Biosciences, Cat#561022<br>APC anti-mouse F4/80, clone RM8, Biolegend, Cat#123116<br>APC/Cyanine7 anti-mouse I-A/I-E, clone M5/114.15.2, Biolegend, Cat#107628<br>Brilliant Violet 421™ anti-mouse CD86, clone GL-1, Biolegend, Cat#105032
Validation	The antibodies and kits for ELISA and Immunohistochemistry were validated according to the manufacturers' websites using suggested working dilutions as described in their Technical Bulletins.

Animals and other organisms

Policy information about studies involving animals; ARRIVE guidelines recommended for reporting animal research

Laboratory animals	Male and female C3H/HeN, C3H/HeJ, C57BL/6, and Ragn12 mice were used for the studies in this paper. See methods section for details.
Wild animals	The study did not involve wild animals.
Field-collected samples	The study did not involve samples collected from the field.
Ethics oversight	All protocols used in this study were approved by the Institutional Animal Care and Use Committee of the University of Chicago and Inotiv.

Note that full information on the approval of the study protocol must also be provided in the manuscript.

Flow Cytometry

Plots

Confirm that:

☒ The axis labels state the marker and fluorochrome used (e.g. CD4-FITC).

☒ The axis scales are clearly visible. Include numbers along axes only for bottom left plot of group (a 'group' is an analysis of identical markers).

☒ All plots are contour plots with outliers or pseudocolor plots.

☒ A numerical value for number of cells or percentage (with statistics) is provided.

Methodology

Sample preparation	Spleen, ileum and colon draining LNs from mice were collected, and digested in DMEM supplemented with 5% FBS, 2.0 mg/mL collagenase D (Sigma Aldrich) and 1.2 mL CaCl2. Single-cell suspensions were prepared by mechanically disrupting the tissues through a cell strainer (70 µm, Thermo Fisher). Splenocytes (4 x 10^6) or cells from LNs (1 x 10^6) were plated in a 96 well-plate. Cells were stained with LIVE/DEAD™ Fixable Aqua Dead Cell Stain Kit (Thermo Fisher), followed by surface staining with antibodies in PBS with 2% FBS, and intracellular staining according to the manufacturer's protocols from eBioscience™ Foxp3/Transcription Factor Staining Buffer Set (Invitrogen).
Instrument	LSR Fortessa flow cytometer (BD Biosciences)
Software	FlowJo 10.8.0
Cell population abundance	Representative cell population abundance are shown in supplementary Fig. S19 and Fig. S20.
Gating strategy	Representative gating strategies are shown in supplementary Fig. S19 and Fig. S20.

☒ Tick this box to confirm that a figure exemplifying the gating strategy is provided in the Supplementary Information.
